# Assessment of diagnostic doses for widely used synthetic pyrethroids (Deltamethrin & Permethrin) in an endemic focus of leishmaniasis in Turkey

**DOI:** 10.1186/s13071-016-1812-y

**Published:** 2016-09-29

**Authors:** Mehmet Karakuş, Yasemen Sarıkaya, Gizem Oğuz, Mert Doğan, Gökhan Ergan, Filiz Günay, Özge Erişöz Kasap, Yusuf Özbel, Bülent Alten

**Affiliations:** 1 Department of Parasitology, Faculty of Medicine, Ege University, Bornova, İzmir Turkey; 2 Department of Biology, Ecology Division, HUESRL-VERG laboratories, Hacettepe University, Ankara, Turkey

**Keywords:** *Phlebotomus*, Insecticide resistance, Pyrethroids, Deltamethrin, Permethrin, Adana, Turkey

## Abstract

**Background:**

*Leishmania* is a group of parasitic flagellated protozoons, which are transmitted by female sand flies and produces health problems in humans and also in wild and domestic animals. So far, 25 *Phlebotomus* and 4 *Sergentomyia* species were recorded in Turkey including proven or possible vectors of *Leishmania* spp. As no single insecticide susceptibility test was conducted targeting the sand flies in Turkey, we aimed to determine the diagnostic dose against two commonly used synthetic pyrethroids (deltamethrin and permethrin) in a hyperendemic area for leishmaniasis.

**Methods:**

Sand flies were collected from villages of Adana in 2–4 September 2013 using Centers for Disease Control and Prevention (CDC) light traps and transferred to the laboratory. The World Health Organisation tube test method was conducted using self-prepared filter papers with different concentrations. In order to determine the diagnostic dose, lethal doses (LD) were calculated by EPA Probit Analysis. Sand flies used in the experiments were dissected, mounted and identified.

**Results:**

For the lowest (0.025 %) and highest dose of permethrin (0.5 %), the mortality rate was recorded as 52.6 % and 100 % by the end of 24-h period and the diagnostic dose was recorded as 0.36 %. The mortality rate for lowest (0.0025 %) and highest (0.05 %) doses of deltamethrin was recorded as 54.8 % and 100 %. The diagnostic dose of deltamethrin was determined as 0.9 %.

**Conclusion:**

An insecticide susceptibility study was conducted in Turkey for the first time and effective doses were determined by calculating the LDs. According to presented results, the wild population of sand flies collected from a hyper-endemic region of Adana Province is still susceptible to deltamethrin and permethrin.

## Background

Phlebotomine sand flies (Diptera: Pyschodidae) are the main vectors of leishmaniasis and a group of viruses called Phleboviruses in the Mediterranean region [1]. In Turkey, two clinical forms of leishmaniasis, namely cutaneous leishmaniasis (CL) and visceral leishmaniasis (VL), are reported. Although the exact incidence of CL is estimated to be much higher than the reported cases, approximately 50,000 cases were reported between 1990–2012 according to the Ministry of Health [2, 3].

Cutaneous leishmaniasis incidence in the Çukurova region was reported previously and the vector species was shown to be *Phlebotomus tobbi* [4, 5]. In Çukurova, the causative agents for CL were previously reported to be *Leishmania infantum* and *L. tropica. Leishmania donovani* and *L. major* were also reported as causative agents in the same area according to a recent investigation of Koltas et al. [6]. Detection of a new phlebovirus in Çukurova highlights the importance of future epidemiological and the entomological studies [7]. Although sand fly fauna of this area and the seasonal dynamics of the vectors has been well documented [8, 9], no study has been performed to measure insecticide resistance of vector sand fly species.

Synthetic pyrethroids has been in use for vector control, in many developed and developing countries since their introduction in 1940s [10]. The use of insecticides for pest or vector control has applied in most malaria endemic countries [11, 12]. In Turkey, vector control programs are mainly targeting mosquitoes; however, no national campaign has been evaluated in order to control Phlebotomine sand flies. Improper, disorganized and uniform usage of insecticides for vector control has led to the development of insecticide resistance in insects and arthropod vectors in tropical and subtropical countries [13]. As the most significant result of the extensive and intensive use of insecticides is the development of resistance against insecticides [14], monitoring of resistance is a necessary element of any medium or large-scale deployment of an insecticidal intervention [15].

The World Health Organization (WHO) exposure kit is widely used for laboratory and field collected sand flies to determine the effective dosages of desired active ingredients [16, 17]. WHO tube test can be performed to measure the insecticide resistance and susceptibility by using insecticide impregnated papers. A standard paper, which is impregnated with different concentrations of insecticides can be obtained from WHO or can be prepared manually by following the previously reported procedure [15].

National control measures against leishmaniasis include disease notification and treatment of patients, but not specific vector control activities. Because of this, effectiveness of the insecticides to those vector sand flies is unknown. Thus, we aimed to determine the diagnostic dosages for widely used synthetic pyrethroids using WHO tube test and wild caught sand flies in an endemic focus of leishmaniasis in Turkey.

## Methods

### Study area and sand fly collection

The sampling was done in villages of Adana (lat 37°15′44.8″N, lon 35°39′24.1″E) between 2–4 September 2013 using Centers for Disease Control and Prevention (CDC) light traps (John W. Hock Company, Gainesville, USA). Light traps were set up at late afternoon and collected by dawn. Alive unfed female sand flies were separated from trap cages using mouth aspirators and transferred to special cones in order to transport to the laboratory, where the bioassay were conducted. Sand flies were kept under stable appropriate conditions (25 ± 2 °C and 70 % ± 10 % relative humidity) during the bioassay period.

### Susceptibility tests

The choice of these two insecticides was justified by their widely usage in Turkey. All plastic equipment needed for the tube test and standard doses of permethrin and deltamethrin are procured from WHOs sales office in Kuala Lumpur, Malaysia. Since there is no standard procedure or baseline insecticide dosage for sand flies described previously in Turkey, different concentrations for both deltamethrin (0.0025 %, 0.005 %, 0.01 %, 0.025 %, 0.05 %) and permethrin (0.025 %, 0.05 %, 0.1 %, 0.25 %, 0.5 %) were prepared by diluting the active ingredient in butanone, which is also obtained from the WHO. Accordingly, control papers were prepared using only butanone. Insecticide impregnated papers used in the present study were prepared using 12 × 15 cm Whatman No. 1 filter papers (Whatman, Madstone, United Kingdom) as noted on WHO Pesticide Evaluation Scheme (WHOPES) [15].

Unfed female sand flies were transferred to exposure tubes with a maximum number of 20 for each tube and were gently transferred to holding tubes after one-hour exposure period. Sugar soaked cotton (10 %) was placed on top of each holding tube and renewed every one hour. Six replicates for each concentration were performed. Control test tubes carrying control papers were also held parallel to each set of tests. The results obtained by each replicate were pooled for diagnostic dose analysis. All bioassays were conducted under stable conditions (25 ± 2 °C and 75 % ± 10 % relative humidity) as stated on WHOPES previously [15].

### Assessment of diagnostic doses

To determine the lethal dose 50, 90 and 99 (LD_50_, LD_90_, LD_99_) for each dilution of the insecticide, mortality was recorded for 1, 3, 6, 12 and 24-h after exposure. Lethal doses and 95 % confidential intervals were calculated using EPA Probit Analysis Software V1.5 [18]. Probit regression lines were illustrated and the Chi-square test for heterogeneity was calculated using the output file of Probit analysis for each insecticide. The slope for LD values were recorded.

### Identification of sand fly species

Following the bioassays, all specimens were transferred to 70 % ethanol for species identification. Sand flies were dissected, mounted and identified using the keys and descriptions presented previously [19–21].

## Results

### Assessment of diagnostic dose for permethrin

**Fig. 1:**
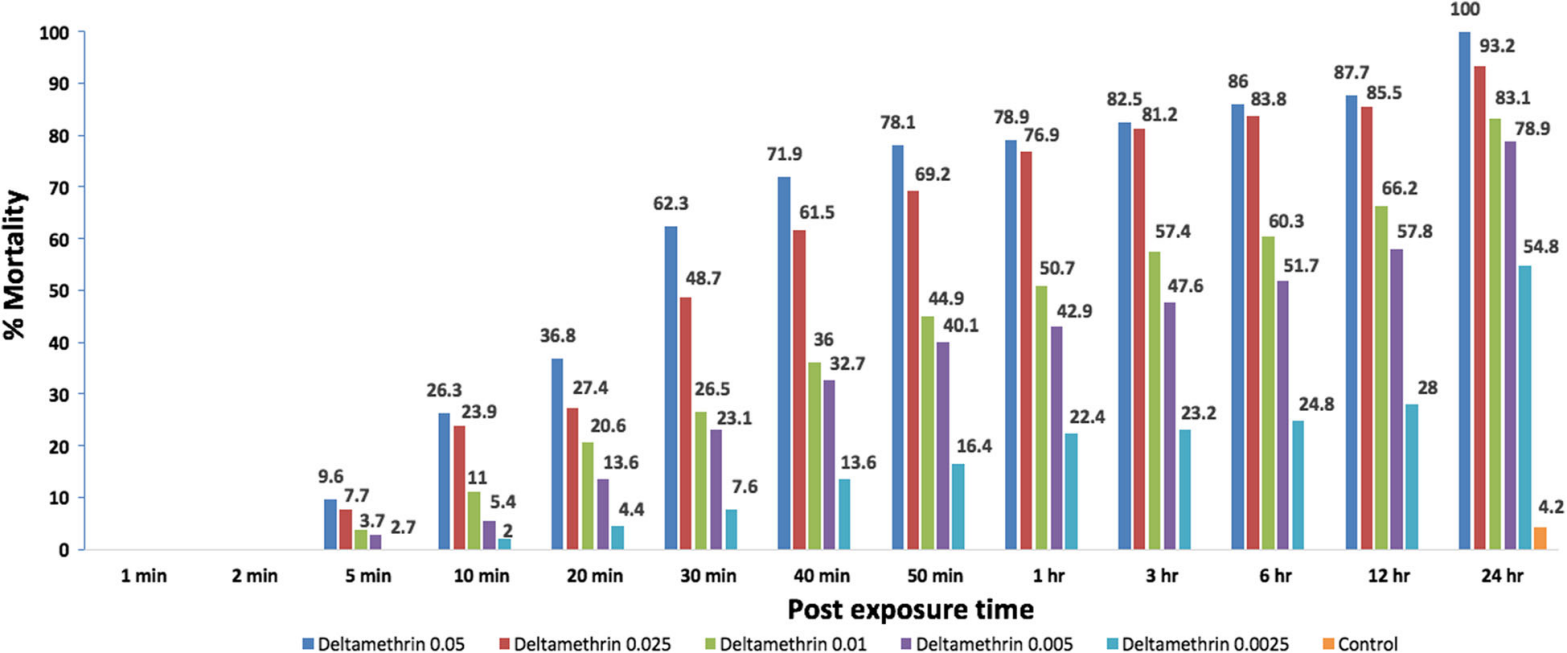
Mortality percentages according to different concentrations of deltamethrin

In total, 882 specimens were exposed to 5 different dilutions of permethrin and diagnostic dose, which was calculated as 0.368 by doubling the LD_99_ value, obtained by Probit analysis. For the lowest dose of permethrin (0.025 %), mortality of 52.6 % was recorded by the end of the 24-h period. For the following doses (0.05 %, 0.1 %, 0.25 % and 0.5 %) mortality rates of 65.3 %, 81.5 %, 92.8 % and 100 %, respectively, were recorded. A total of 113 specimens were used in the control group and 3.5 % mortality was observed. According to WHO, all bioassays were accepted as valid since the mortality of the control group observed to be lower than 20 % (Fig. 1).

### Assessment of diagnostic dose for deltamethrin

**Fig. 2:**
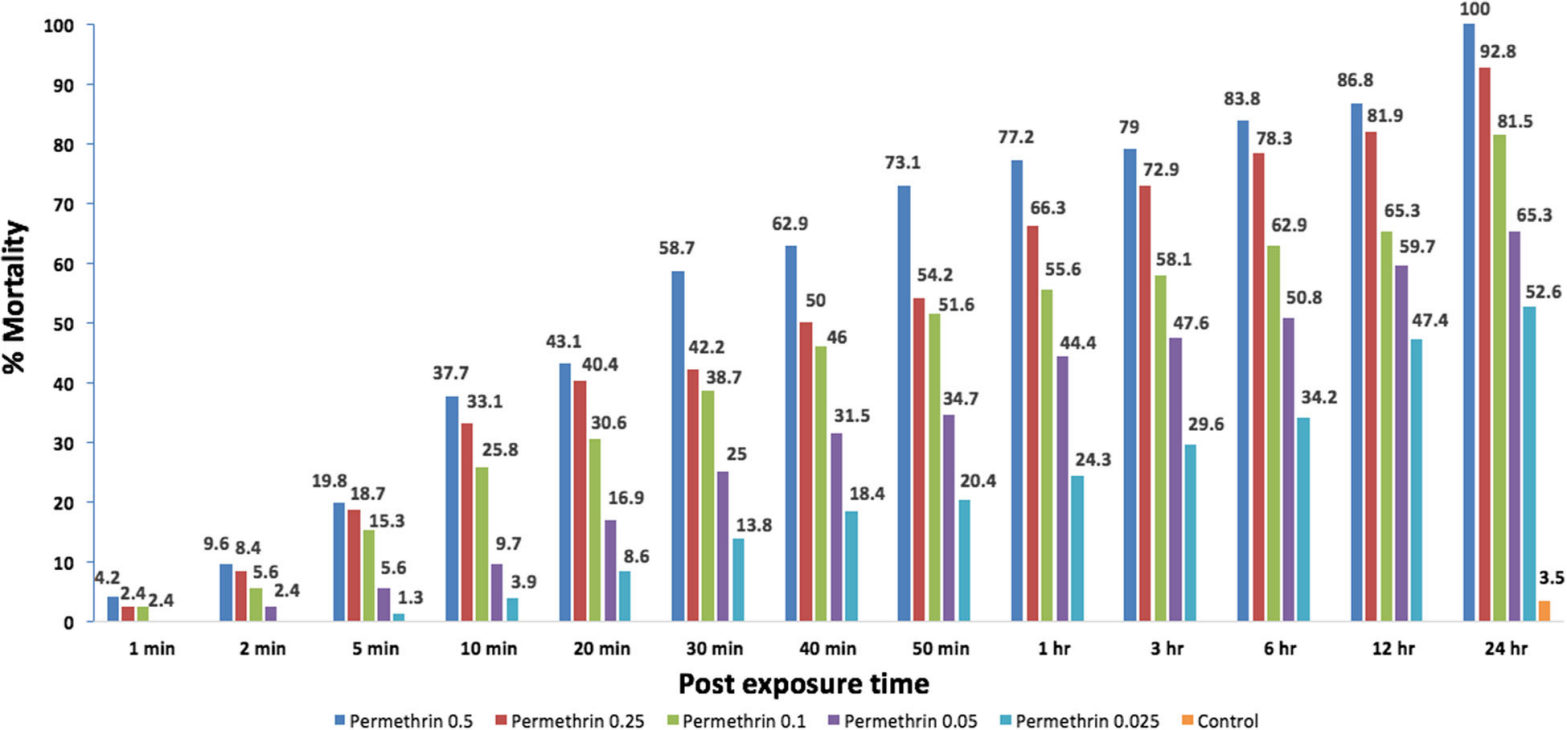
Mortality percentages according to different concentrations of permethrin

A total of 846 specimens were exposed to 5 different doses of deltamethrin, and the diagnostic dose was noted as 0.9 % by doubling the LD_99_ value. Mortality for the lowest dose of deltamethrin (0.0025 %) was found to be 54.8 % by the end of the 24-h period. For the following doses of deltamethrin (0.005 %, 0.01 %, 0.025 %, 0.05 %), mortalities were noted as 78.9 %, 83.1 %, 93.2 % and 100 %, respectively. The control group was conducted using 118 specimens and 4.2 % mortality was observed by the end of 24-h period. (Fig. 2). LD values, diagnostic doses, *P*-values and Chi-square data were shown in table (Table 1).

**Table 1:** Probit analysis results of deltamethrin and permethrin

	Deltamethrin	Permethrin
Number exposed	882	846
LD_50_ % (95 % CI^a^)	0.002 (0.002–0.003)	0.007 (0.001–0.015)
LD_90_ % (95 % CI^a^)	0.027 (0.019–0.044)	0.238 (0.152–0.480)
LD_99_ % (95 % CI^a^)	0.184 (0.094–0.537)	0.450 (0.312–1.702)
Diagnostic dose	0.368	0.9
*χ* ^2^	6.614	1.943
*P*-value	0.079	0.064

^a^Upper and Lower Limits with 95 % confidence intervals

### Assessment of diagnostic doses for *Phlebotomus tobbi*

**Fig. 3:**
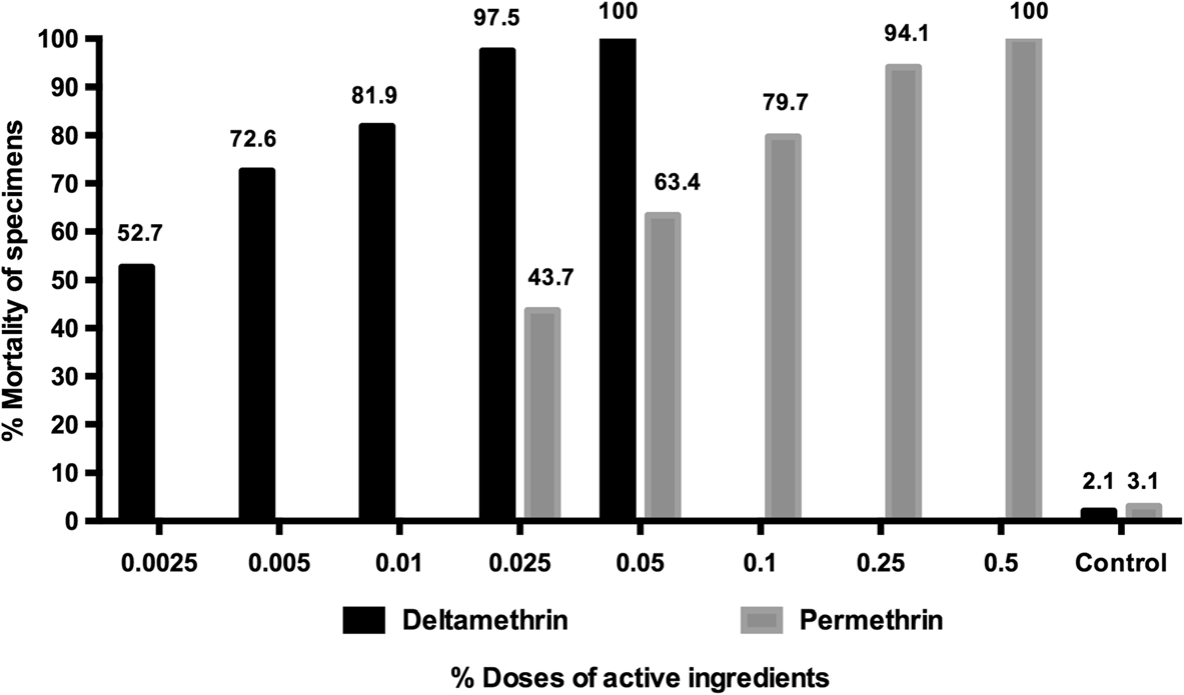
Mortality percentages of *P. tobbi* according to different concentrations of deltamethrin and permethrin

Of the 1,959 specimens tested, 948 of them were classified as *P. tobbi*. Results of the experiments were evaluated separately and Probit analysis was done particularly to *P. tobbi*. A total of 948 specimens were exposed to 5 different doses of both deltamethrin and permethrin and diagnostic doses were noted as 0.34 % and 0.75 % by doubling the LD_99_ value, respectively. Mortality was found to be 52.7 % and 43.7 % by the end of 24-h period for the lowest doses of deltamethrin (0.0025 %) and permethrin (0.025 %). For the highest tested doses of deltamethrin (0.05 %) and Permethrin (0.5 %), 100 % death rate was observed by the end of 24-h period. (Fig. 3).

### Sand fly species used in the experiments

In total, 1,959 (1,728 in the exposure tests and 231 in the control group) sand flies used in the bioassays were identified and 5 species belonging to two genera, *Phlebotomus* [*P. tobbi* 48.3 %, *P. papatasi* 23.1 %, *P. perfiliewi* 18.4 % and 3.1 % *P. major* (*s.l*.)] and *Sergentomyia* (7.1 % *S. dentata)* were identified.

## Discussion

Phlebotomine sand flies are the main vectors of *Leishmania* spp. and also play a role in the transmission of arboviruses (Phleboviruses) and bacteria (*Bartonella bacilliformis*) to both humans and animals. So far, 25 *Phlebotomus* and four *Sergentomyia* species were recorded in Turkey including proven or possible vectors of leishmaniasis and other sand fly borne diseases [4, 8, 22]. The national leishmaniasis control program is currently covers patient treatment free-of-charge, but there is no vector control program was conducted in Turkey. However, indoor residual spraying has mainly been used for malaria control programs in most of the regions in the country and it affects other insects including sand flies.

The resistance status of commonly used chemicals to mosquitoes was studied in Çukurova plain since 1959 and developed resistance was shown in natural mosquito populations of Adana province [10, 23]. Due to sharing the same habitat with mosquitoes, the development of insecticide resistance in sand flies is inevitable. Diagnostic dose analyses were also done with the proven vector species *P. tobbi*. According to our results, LD values of both insecticides were noted to be lower in *P. tobbi* specimens. Different sand fly species do not necessarily share the same resistance mechanism, nor do they exhibit the same resistance patterns. In the present study, the diagnostic dose of deltamethrin (0.34 %) and permethrin (0.75 %) were evaluated between 95 % confidential intervals by EPA Probit Analysis.

The suitability of WHO bioassay for determining the insecticide susceptibility is widely accepted on sand flies as well as on other insect species [24–26]. Another tool for screening the susceptibility of sand flies to desired insecticides is the CDC bottle bioassay tests [15, 27]. There are several concentrations and methods, applied to measure the insecticide susceptibility, but no standard dose or method was determined for sand flies [26, 28]. In previous studies, researchers modified the CDC bottle bioassay by using different size of glass bottles [29, 30]. This resulted in different percentages of mortality. Denlinger et al. [29] reported that mortality percentage was higher in small glass bottles, in comparison with the big glass bottles. Since the method used was not universal, the results obtained in these studies cannot be compared between different labs. In the present study, no modifications were made on WHO tube test equipment’s in order to compare the results with other laboratories that using the same standards of WHO.

The characteristics of the pyrethroids used in this study are known to be different. Type I pyrethroids including permethrin are more likely to cause knockdown in a very short time period, while Type II pryethroids are more likely to cause mortality [31]. In the present study we noted that LD_50_ value of permethrin was almost 3 times higher than the LD_50_ value of deltamethrin (Table 1). This difference in LD values does not arise because of resistance, but most likely because it is a different class of insecticide. Previous studies correlate with our findings, which state that, high concentration of permethrin is needed to cause the same percentage of mortality compared to deltamethrin [29, 32, 33].

As a results of exposure to pyrethroids and DDT, shedding of the legs is an important sub-lethal effect in sand flies. This effect of prethroids were observed in different studies using deltamethrin, permethrin and lambda-cyhalothrin [34]. It was previously suggested that, shedding of legs will reduce the blood-feeding behavior of the sand flies and will help to control leishmaniasis [34]. However, contradictory results were obtained by Denlinger et al. [29], who suggested that sand flies that have shed their legs are still capable of feeding on anesthetized mice. In the present study, shedding of legs was observed in each concentration of deltamethrin and permethrin. In the lowest concentration group, sand flies with shed legs survived by the end of 24-h period. Rogers et al. [35] reported that the infection with *Leishmania* will manipulate the sand fly to increase their biting frequency. It is clear that ineffective doses will cause sand flies to shed their legs but this will not obstruct their role in the transmission of *Leishmania*.

As stated on WHO and CDC manuals for insecticide tests, only unfed female specimens can be used in the experiments [15]. In a previous study, researchers reported that relative toxicity of the insecticides can be assessed by using male specimens in the insecticide susceptibility experiments [30]. Because of the physiological features and body size changes according to the gender of insects, we decided to use only non-blood-fed female specimens in each experiments as well as in control group. The use of different methods in the insecticide susceptibility tests makes it harder to compare and/or combine the data obtained from different research facilities.

The results of previous studies have shown that sand flies are less likely to develop resistance and are still susceptible to the majority of the available insecticides, which are actively in use [36–38]. Molecular approaches were also evaluated to test the pyrethroid resistance and failed to detect resistance among wild populations [39]. Our findings were similar to those previous insecticide susceptibility studies. Pyrethroid resistance in mosquitos were demonstrated in the study area previously, but the findings of the present study suggest that the sand fly populations of the study area are still susceptible to these insecticides [40].

### Study limitations

Because there is no published study about the baseline dose for pyrethroid insecticides in the study area, we performed the diagnostic dose assessment tests using the standard doses for deltamethrin and permethrin, which evaluated by WHO. Because of the species identification can only made after the bioassays in wild-caught experiments, we could not assess the LD values particular to pathogenic species and LD values couldn’t be determined on species level.

## Conclusion

This is the first study that aims to assess the diagnostic dose for commonly used synthetic pyrethroids in an leishmaniasis endemic area in Turkey. Improper, disorganized and uniform usage of the insecticides for vector control will lead to the development of insecticide resistance in vector species. Routine monitoring of insecticide resistance in the natural populations of vectors is necessary and will helps to detect early resistance and improve effectiveness of operational control strategies. The data obtained from this study will help to national vector control programs to use appropriate and effective insecticide applications. Further studies are needed to evaluate diagnostic doses for *P. papatasi* and for other possible vector species in the study area.

## Abbreviations

CDC: Centers for disease control and Pprevention
CL: Cutaneous leishmaniasis
LD: Lethal dose
VL: Visceral leishmaniasis
WHO: World Health Organization
WHOPES: World Health Organization Pesticide Evaluation Scheme
